# Autoimmune comorbidity in type 1 diabetes and its association with metabolic control and mortality risk in young people: a population-based study

**DOI:** 10.1007/s00125-024-06086-8

**Published:** 2024-01-22

**Authors:** John Samuelsson, Rebecka Bertilsson, Erik Bülow, Sanna Carlsson, Sanna Åkesson, Björn Eliasson, Ragnar Hanas, Karin Åkesson

**Affiliations:** 1 Department of Paediatrics, Ryhov County Hospital, Jönköping, Sweden; 2 Department of Biomedical and Clinical Sciences, Linköping University, Linköping, Sweden; 3 Centre of Registers in Region Västra Götaland, Gothenburg, Sweden; 4 Department of Orthopaedics, Institute of Clinical Sciences, Sahlgrenska Academy, University of Gothenburg, Gothenburg, Sweden; 5 The Sahlgrenska Academy, Institute of Medicine, University of Gothenburg, Gothenburg, Sweden; 6 Department of Medicine, Sahlgrenska University Hospital, Gothenburg, Sweden; 7 The Sahlgrenska Academy, Institute of Clinical Sciences, University of Gothenburg, Gothenburg, Sweden; 8 Department of Paediatrics, NU Hospital Group, Uddevalla, Sweden

**Keywords:** Adolescents, Autoimmune comorbidity, Children, HbA_1c_, Metabolic control, Mortality, Quality register, Type 1 diabetes, Young adults

## Abstract

**Aims/hypothesis:**

This register-based study aimed to describe autoimmune comorbidity in children and young adults from type 1 diabetes onset, and to investigate whether such comorbidity was associated with a difference in HbA_1c_ or mortality risk compared with children/young adults with type 1 diabetes without autoimmune comorbidity.

**Methods:**

A total of 15,188 individuals from the Swedish National Diabetes Register, registered with type 1 diabetes before 18 years of age between 2000 and 2019, were included. Five randomly selected control individuals from the Swedish population (Statistics Sweden) were matched to each individual with type 1 diabetes (*n*=74,210 [346 individuals with type 1 diabetes were not found in the Statistics Sweden register at the date of type 1 diabetes diagnosis, so could not be matched to control individuals]). The National Patient Register was used to attain ICD-10 codes on autoimmune diseases and the Cause of Death Register was used to identify deceased individuals.

**Results:**

In the total type 1 diabetes cohort, mean±SD age at onset of type 1 diabetes was 9.5±4.4 years and mean disease duration at end of follow-up was 8.8±5.7 years. Of the individuals with type 1 diabetes, 19.2% were diagnosed with at least one autoimmune disease vs 4.0% of the control group. The HRs for comorbidities within 19 years from onset of type 1 diabetes were 11.6 (95% CI 10.6, 12.6) for coeliac disease, 10.6 (95% CI 9.6, 11.8) for thyroid disease, 1.3 (95% CI 1.1, 1.6) for psoriasis, 4.1 (95% CI 3.2, 5.3) for vitiligo, 1.7 (95% CI 1.4, 2.2) for rheumatic joint disease, 1.0 (95% CI 0.8, 1.3) for inflammatory bowel disease, 1.0 (95% CI 0.7, 1.2) for systemic connective tissue disorder, 1.4 (95% CI 1.1, 1.9) for uveitis, 18.3 (95% CI 8.4, 40.0) for Addison’s disease, 1.8 (95% CI 0.9, 3.6) for multiple sclerosis, 3.7 (95% CI 1.6, 8.7) for inflammatory liver disease and 19.6 (95% CI 4.2, 92.3) for atrophic gastritis. Autoimmune disease in addition to type 1 diabetes had no statistically significant effect on HbA_1c_ or mortality risk.

**Conclusions/interpretation:**

To our knowledge, this is the first comprehensive study where young individuals with type 1 diabetes were followed regarding development of a wide spectrum of autoimmune diseases, from onset of type 1 diabetes. In this nationwide and population-based study, there was already a high prevalence of autoimmune diseases in childhood, especially coeliac and thyroid disease. The presence of autoimmune comorbidity did not have a statistically significant effect on metabolic control or mortality risk.

**Graphical Abstract:**

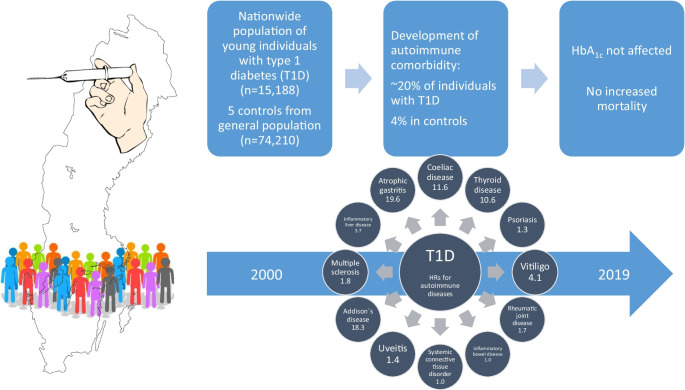


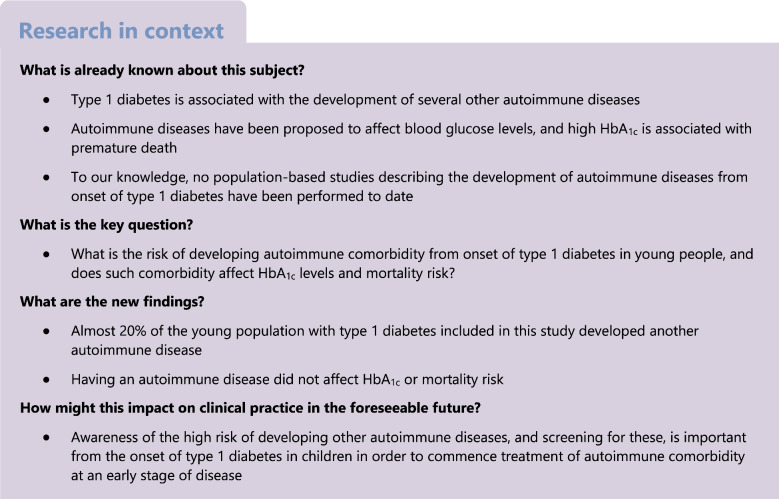


## Introduction

Type 1 diabetes is the second-most prevalent chronic illness affecting children in Sweden, with roughly 900 new cases diagnosed each year. The incidence of type 1 diabetes in Sweden is exceptionally high, with a rate of 43 cases per 100,000 person-years at risk in 2019, making it the world’s second-highest incidence after Finland [1]. Although the pathophysiology of type 1 diabetes is not fully understood, it is believed to be partially attributed to the autoimmune destruction of pancreatic beta cells, resulting in absolute insulin deficiency and damage to vital organs [2]. Associations between type 1 diabetes and other autoimmune diseases (AIDs) are well-documented, with autoimmune thyroid disease, coeliac disease, atrophic gastritis, vitiligo, hyperthyroidism and Addison’s disease being the most prevalent [3]. The link between type 1 diabetes and these diseases is due, in part, to genetic susceptibility [4–6]. In a large American study involving both children and adults, 27% of individuals with type 1 diabetes were diagnosed with at least one additional AID [7]. Similar results were found in a Finnish study that reported a prevalence of 22.8% of AIDs in adults with type 1 diabetes. Late onset of type 1 diabetes was associated with an increased risk of hypothyroidism, while younger age at onset was associated with an increased risk of coeliac disease [8]. The prevalence of AIDs increases with age and is more common in female individuals [9]. Previous studies conducted in Sweden showed that 7–10% of individuals with type 1 diabetes had coeliac disease [10, 11], with the highest risk of developing the condition within the first 2 years after diagnosis [10]. Individuals with both type 1 diabetes and coeliac disease have an increased risk of diabetic microvascular complications [12].

AIDs can affect blood glucose levels directly through physiological means or indirectly through medications, such as high-dose corticosteroids [13]. Good metabolic control in childhood and adolescence is critical, as it impacts future metabolic control, risk of complications, and premature mortality [14, 15]. HbA_1c_ levels are closely linked to premature death in adults [16].

To our knowledge, no paediatric population-based studies have been conducted to date that have examined the full spectrum of AID development from type 1 diabetes onset. Existing studies have been conducted on a limited population, a limited number of AIDs, or in adults [4, 7–9, 13, 17, 18]. By leveraging a nationwide population-based register that contains data on glycaemic control and clinical variables, linked with population and health data registers in Sweden, we have a unique opportunity to examine autoimmune comorbidity and its association with clinical factors in a young Swedish population with type 1 diabetes. The objective of our study was to explore the risk of autoimmune comorbidity in children, adolescents and young adults with type 1 diabetes in Sweden and to investigate whether such comorbidity was linked to differences in HbA_1c_ levels or mortality risk.

## Methods

**Fig. 1:**
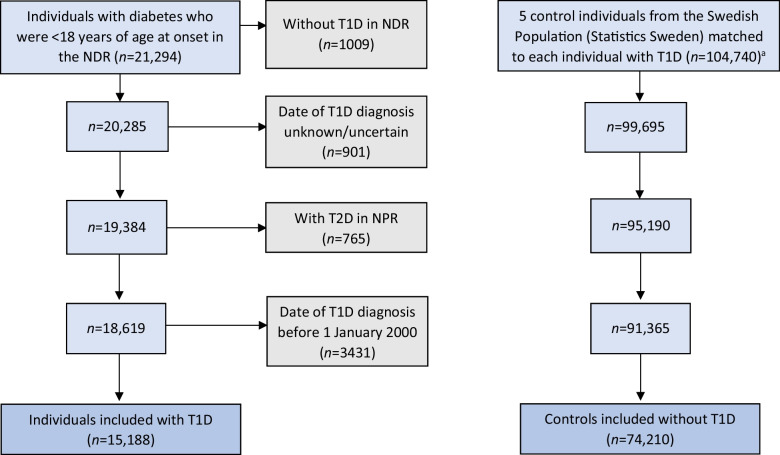
Flowchart of selection of participants. ^a^346 individuals with type 1 diabetes were not found in the Statistics Sweden register at the date of type 1 diabetes diagnosis and, therefore, could not be matched to five control individuals. T1D, type 1 diabetes; T2D, type 2 diabetes

In this study, all 21,294 individuals registered with type 1 diabetes between 2000 and 2019 were included from the paediatric part (former Swediabkids) of the National Diabetes Register (NDR), from the date of entrance into the register. The NDR was founded in 2000 and, since 2007, it has an almost complete coverage (97.5% year 2017) of children and adolescents diagnosed with type 1 diabetes below 18 years of age in Sweden. Data are reported directly from diabetes clinics to the register [1]. After selection, 15,188 individuals were included in the study (Fig. 1). Date of diagnosis of type 1 diabetes, baseline data and information on HbA_1c_ were retrieved from the NDR. To act as a control group, five individuals were randomly selected from the Swedish population (Statistics Sweden) and matched to each individual with type 1 diabetes (exact matching without replacement by age, sex and county of residence), rendering 74,210 control individuals (note that 346 individuals with type 1 diabetes were not found in the Statistics Sweden register at the date of diagnosis of type 1 diabetes and, therefore, could not be matched to five control individuals). Sex of the included individuals was registered in the Swedish population register, with data obtained using personal identity numbers. Data on race, ethnicity and gender were not available in this study. To attain ICD-10 codes (https://icd.who.int/browse10/2019/en) for AIDs, this population was linked with the Swedish National Patient Register (NPR). Since 1987, this register has included all in-patient care in Sweden, and since 2001 it has also covered outpatient visits, including day surgery and psychiatric care from both private and public caregivers. Primary care is not yet covered in the NPR [19]. Data were obtained for the period 1997–2019. For details regarding diagnosis classification (ICD-10 codes), see Table 1. The Swedish Prescribed Drug Register (SPDR) was used to ascertain prescription of insulin to included individuals with type 1 diabetes. This register, started in July 2005, includes all drugs collected with prescription at pharmacies and includes data on the individual, the drug (anatomical therapeutic chemical [ATC]-code), the prescription, characteristics of the workplace where the drug was prescribed, and profession and specialty of the prescriber [20]. Finally, the cohort was linked with The Swedish Cause of Death Register (CDR) [21], in order to identify deceased individuals. The NPR, SPDR and CDR are held by The National Board of Health and Welfare in Sweden. The linkage of individuals with type 1 diabetes and control individuals was done by Statistics Sweden and The National Board of Health and Welfare in Sweden, using personal identification numbers [22].

**Table 1:** ICD-10 codes used for the identification of AIDs

Disease	The Swedish NPR (ICD-10 code)
Coeliac disease	K90.0
Thyroid disease	E03.8, E03.9, E05.8, E05.9, E06.3
Psoriasis	L40
Vitiligo	L80
Rheumatic joint diseases	M05–M08
Inflammatory bowel disease	K50–K51
Systemic connective tissue disorder	M30-M36
Uveitis	H20, H22.1
Addison’s disease	E27.1, E27.2
Multiple sclerosis	G35
Inflammatory liver disease	K75.4
Atrophic gastritis	K29.4

The study protocol was approved by the Swedish Ethical Review Authority (Dnr: 2019-03600).

### Statistical analysis

Baseline characteristics for continuous variables are presented as mean±SD, and categorical variables are presented as percentages and frequencies. Variance analysis was made using Kruskal–Wallis test for numeric variables and χ^2^ test for categorical variables. A *p* value <0.05 was considered significant. The Kaplan–Meier method was used to estimate cumulative risks and Cox regression was used to estimate HRs with 95% CIs. HRs were adjusted for age. The cumulative risks were visualised reversing the Kaplan–Meier curve. Events occurring before the index date (date of diagnosis of type 1 diabetes) are presented at time zero, sometimes leading to an elevated starting point on the *y*-axis of plots. The individuals in the cohort were followed until death or administrative censoring (end of follow-up 31 December 2019), whichever came first. Smoothed conditional means were used to illustrate the association between diabetes duration and HbA_1c_ by diagnosis group. The data management and analysis were primarily conducted in SAS version 9.4 (SAS Institute, Cary NC, USA) and R version 4.0.2 (R Core Team, Vienna Austria).

## Results

Mean±SD age at onset of type 1 diabetes was 9.5±4.4 years and mean±SD duration of type 1 diabetes was 8.8±5.7 years. Individuals with an additional AID were, on average, slightly younger at diabetes onset (mean±SD 9.2±4.5 years vs 9.5±4.4 years) than individuals with type 1 diabetes only and had a longer duration of type 1 diabetes at the end of follow-up (mean±SD 10.5±5.5 years vs 8.5±5.7 years). There was no clinically significant baseline difference in children with type 1 diabetes vs children with type 1 diabetes and an additional AID regarding HbA_1c_ and BMI at onset of type 1 diabetes (Table 2). The prevalence of the various AIDs at the onset of type 1 diabetes is shown in Table 3. In individuals with type 1 diabetes, 19.2% (*n*=2916) were diagnosed with at least one additional AID during the study period (in total 3312 AIDs), compared with 4.0% (*n*=2996) of the control individuals (3259 AIDs) (see Table 4). In total 44.6% of the population with type 1 diabetes were female but there was a preponderance of female individuals with type 1 diabetes and at least one additional AID (56.7%). In total, 24.4% of the girls and 15.0% of the boys developed at least one additional AID. In individuals with type 1 diabetes 2.0% developed two AIDs and 0.3% developed three or more AIDs (Table 4). The most common additional AID in type 1 diabetes individuals at end of follow-up was coeliac disease, followed by thyroid disease, psoriasis, vitiligo, rheumatic joint disease, inflammatory bowel disease, systemic connective tissue disorders, uveitis, Addison’s disease, multiple sclerosis, inflammatory liver disease and atrophic gastritis (Table 4).

**Table 2:** Baseline characteristics of individuals with type 1 diabetes and control participants with and without AID

Characteristic	T1D	T1D+AID	Control	Control+AID
*n*	12,272	2916	71,214	2996
Sex, female	5122 (42)	1652 (57)	31,423 (44)	1662 (55)
Age at inclusion, years	9.5±4.4^a^	9.2±4.5^a^	9.5±4.4	10.5±4.1
Duration of follow-up, years	8.5±5.7^b^	10.5±5.5^b^	8.7±5.7	10.8±5.5
HbA_1c_, mmol/mol^c^	47.6±9.9	48.2±10.6	–	–
HbA_1c_, %^c^	6.5±0.9	6.6±1.0	–	–
BMI, kg/m^2^	18.6±3.3	18.3±3.2	–	–

Data are shown as mean±SD or *n *(%)

^a^Age at inclusion equates to age at type 1 diabetes onset

^b^Duration of follow-up equates to diabetes duration

^c^First measured value after 90 days and within 1 year from type 1 diabetes onset

T1D, type 1 diabetes

**Table 3:** Prevalence of AIDs in control individuals and individuals with type 1 diabetes at inclusion in study or onset of diabetes, respectively

AID	Control individuals			Individuals with T1D		
	Overall	Female individuals	Male individuals	Overall	Female individuals	Male individuals
*n*	74,210	33,085	41,125	15,188	6774	8414
Coeliac disease	387 (0.5)	217 (0.7)	170 (0.4)	274 (1.8)	150 (2.2)	124 (1.5)
Thyroid disease	129 (0.2)	70 (0.2)	59 (0.1)	88 (0.6)	50 (0.7)	38 (0.5)
Psoriasis	119 (0.2)	50 (0.2)	69 (0.2)	26 (0.2)	11 (0.2)	15 (0.2)
Vitiligo	51 (0.1)	31 (0.1)	20 (0.0)	25 (0.2)	11 (0.2)	14 (0.2)
Rheumatic joint disease	139 (0.2)	82 (0.2)	57 (0.1)	34 (0.2)	20 (0.3)	14 (0.2)
Inflammatory bowel disease	75 (0.1)	34 (0.1)	41 (0.1)	20 (0.1)	4 (0.1)	16 (0.2)
SCTD	147 (0.2)	65 (0.2)	82 (0.2)	29 (0.2)	14 (0.2)	15 (0.2)
Uveitis	51 (0.1)	28 (0.1)	23 (0.1)	11 (0.1)	5 (0.1)	6 (0.1)
Addison’s disease	0 (0.0)	0 (0.0)	0 (0.0)	2 (0.0)	0 (0.0)	2 (0.0)
Multiple sclerosis	0 (0.0)	0 (0.0)	0 (0.0)	0 (0.0)	0 (0.0)	0 (0.0)
Inflammatory liver disease	1 (0.0)	0 (0.0)	1 (0.0)	1 (0.0)	0 (0.0)	1 (0.0)
Atrophic gastritis	0 (0.0)	0 (0.0)	0 (0.0)	0 (0.0)	0 (0.0)	0 (0.0)
Total number of autoimmune diagnoses	1099	577	522	510	265	245

Data are shown as *n* (%) or *n*

SCTD, systemic connective tissue disorder; T1D, type 1 diabetes

**Table 4:** Prevalence of AIDs in control individuals and individuals with type 1 diabetes at end of follow-up

AID	Control individuals			Individuals with T1D			HR (95% CI)
	Overall	Female individuals	Male individuals	Overall	Female individuals	Male individuals	
*n*	74,210	33,085	41,125	15,188	6774	8414	
One AID	2765 (3.7)	1524 (4.6)	1241 (3.0)	2562 (16.9)^***^	1436 (21.2)	1126 (13.4)	–
Two AIDs	204 (0.3)	121 (0.4)	83 (0.2)	311 (2.0)^***^	192 (2.8)	119 (1.4)	–
Three or more AIDs	27 (0.0)	17 (0.1)	10 (0.0)	43 (0.3)^***^	24 (0.4)	19 (0.2)	–
Coeliac disease	712 (1.0)	429 (1.3)	283 (0.7)	1604 (10.6)	864 (12.8)	740 (8.8)	11.55 (10.58, 12.62)
Thyroid disease	518 (0.7)	377 (1.1)	141 (0.3)	1072 (7.1)	717 (10.6)	355 (4.2)	10.61 (9.56, 11.79)
Psoriasis	498 (0.7)	225 (0.7)	273 (0.7)	130 (0.9)	68 (1.0)	62 (0.7)	1.28 (1.06, 1.55)
Vitiligo	134 (0.2)	70 (0.2)	64 (0.2)	112 (0.7)	46 (0.7)	66 (0.8)	4.09 (3.19, 5.26)
Rheumatic joint disease	309 (0.4)	176 (0.5)	133 (0.3)	109 (0.7)	72 (1.1)	37 (0.4)	1.73 (1.39, 2.15)
Inflammatory bowel disease	431 (0.6)	194 (0.6)	237 (0.6)	88 (0.6)	30 (0.4)	58 (0.7)	1.00 (0.80, 1.26)
SCTD	385 (0.5)	220 (0.7)	165 (0.4)	75 (0.5)	42 (0.6)	33 (0.4)	0.95 (0.74, 1.22)
Uveitis	222 (0.3)	98 (0.3)	124 (0.3)	65 (0.4)	30 (0.4)	35 (0.4)	1.44 (1.09, 1.90)
Addison’s disease	8 (0.0)	5 (0.0)	3 (0.0)	30 (0.2)	8 (0.1)	22 (0.3)	18.33 (8.40, 39.98)
Multiple sclerosis	28 (0.0)	20 (0.1)	8 (0.0)	10 (0.1)	6 (0.1)	4 (0.0)	1.76 (0.86, 3.63)
Inflammatory liver disease	12 (0.0)	5 (0.0)	7 (0.0)	9 (0.1)	3 (0.0)	6 (0.1)	3.68 (1.55, 8.73)
Atrophic gastritis	2 (0.0)	1 (0.0)	1 (0.0)	8 (0.1)	6 (0.1)	2 (0.0)	19.61 (4.16, 92.34)
Total number of autoimmune diagnoses	3259	1820	1440	3312	1938	1419	–

Data are shown as *n* (%) or *n*

^***^p<0.01 vs overall control population

SCTD, systemic connective tissue disorder; T1D, type 1 diabetes

**Fig. 2:**
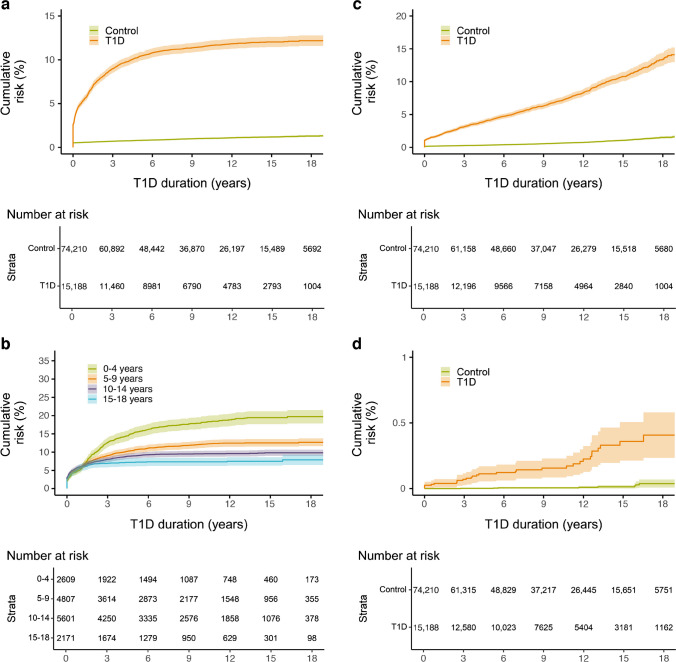
(**a**, **b**) Cumulative risk for developing coeliac disease during the study period among individuals with type 1 diabetes and control individuals (**a**), stratified into age groups at onset of type 1 diabetes (**b**). (**c**, **d**) Cumulative risk for developing thyroid disease (**c**) and Addison’s disease (**d**) during the study period among individuals with type 1 diabetes and control individuals. Shaded areas represent 95% CI. T1D, type 1 diabetes

**Fig. 3:**
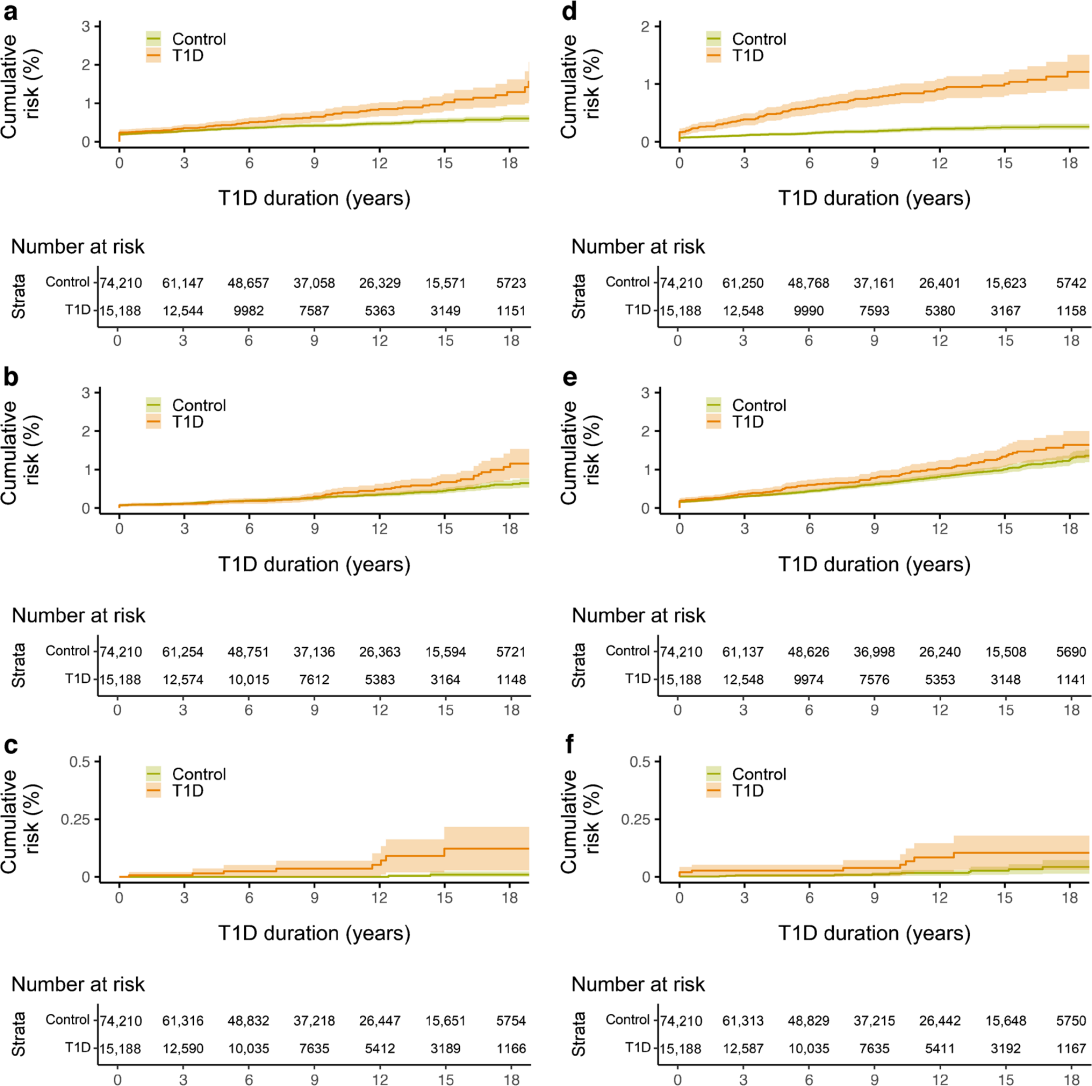
Cumulative risk for developing rheumatic joint disease (**a**), uveitis (**b**), atrophic gastritis (**c**), vitiligo (**d**), psoriasis (**e**) and inflammatory liver disease (**f**) during the study period among individuals with type 1 diabetes and control individuals. Shaded areas represent 95% CI. T1D, type 1 diabetes

The hazard of coeliac disease was, both clinically and statistically, significantly higher in children diagnosed with type 1 diabetes at a younger age (*p*<0.0001), with most cases evident in the lowest age stratum (0–4 years); cumulative risk: ~20 %). Although the incidence was highest in the first 5 years after type 1 diabetes diagnosis, there were still children in the youngest age stratum diagnosed with coeliac disease after having type 1 diabetes for more than 15 years (Fig. 2a,b). Thyroid disease was more common in the type 1 diabetes population than in the control population, even in young children. The excess risk of thyroid disease in the type 1 diabetes population increased with increasing age, starting from diabetes onset (Fig. 2c). Hypothyroidism was the most prevalent thyroid disease. The hazard of Addison’s disease was higher in those with type 1 diabetes than in the control population (Fig. 2d), but few individuals were diagnosed in total. The risk of Addison’s disease was very low among children with a short diabetes duration. Rheumatic joint disease was more prevalent in the type 1 diabetes population (Fig. 3a). The incidence of uveitis was low for the first 9 years after diagnosis but increased thereafter, being more pronounced in the type 1 diabetes population (Fig. 3b). Very few individuals were diagnosed with atrophic gastritis (Fig. 3c) and few were diagnosed with vitiligo (Fig. 3d). Psoriasis was more common in individuals with type 1 diabetes than in the control population (Fig. 3e); however, although statistically significant (*p*=0.013), no clinically significant difference in the development of the disease was found. Inflammatory liver disease (Fig. 3f) was found in only nine individuals in the type 1 diabetes population vs 12 people in the control population (*p*=0.0016). In a Kaplan–Meier analysis, no statistically significant difference in the development of inflammatory bowel disease, multiple sclerosis or systemic connective tissue disorder was found (data not shown). For corresponding HRs for each disease, see Table 4.

### HbA_1c_

**Fig. 4:**
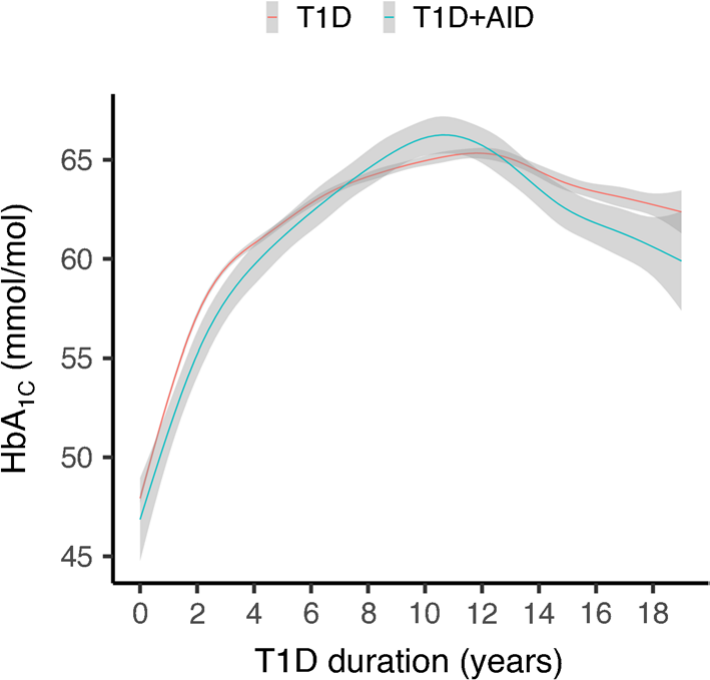
Locally estimated scatterplot smoothing (LOESS) plot of yearly mean HbA_1c_ from onset and from follow-up visits up to a type 1 diabetes duration of 19 years, comparing individuals with type 1 diabetes only with individuals with type 1 diabetes and other AIDs. Shaded areas represent 95% CI. T1D, type 1 diabetes

When comparing individuals with type 1 diabetes with those with type 1 diabetes and at least one additional AID, no difference in HbA_1c_ could be seen during the study period (Fig. 4). The same was true when analysing each AID separately (data not shown). By the end of the follow-up period, the mean HbA_1c_ was 62 mmol/mol (7.8%).

### Mortality

**Fig. 5:**
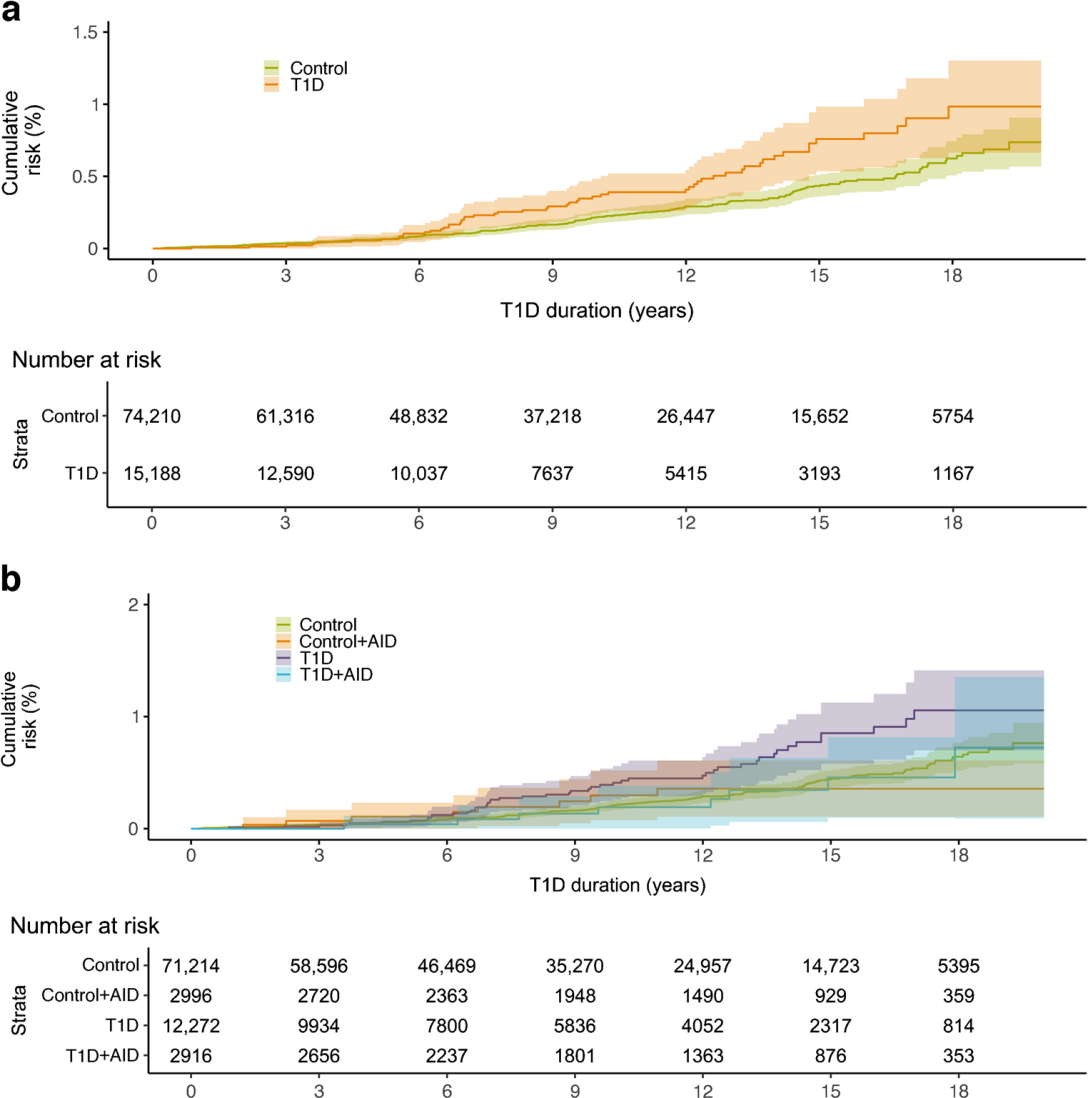
(**a**) Mortality risk in individuals with type 1 diabetes compared with control individuals. (**b**) Mortality risk stratified by presence of another AID(s) in individuals with type 1 diabetes and control individuals. T1D, type 1 diabetes

Individuals with type 1 diabetes had an increased risk of premature death compared with control individuals (Fig. 5a), with an HR of 1.57 (95% CI 1.16, 2.12), although the CIs in the Kaplan–Meier analysis overlapped. Individuals with an additional AID did not have an increased mortality risk when compared with those with type 1 diabetes but no other AID (Fig. 5b). The corresponding HR for individuals with type 1 diabetes but no other AID was 1.74 (95% CI 1.26, 2.40) and for individuals with type 1 diabetes and an additional AID it was 0.95 (95% CI 0.47, 1.92).

## Discussion

This is a comprehensive nationwide case–control study conducted in Sweden, a country with one of the highest incidences of type 1 diabetes in the world. The study is population-based and utilises register data. To the best of our knowledge, this is the first investigation to explore the risk of additional AIDs in individuals with type 1 diabetes from the onset of type 1 diabetes, with a mean duration of 8.8 years in the total type 1 diabetes cohort (maximum duration: 19 years). The study encompasses a wide range of AIDs.

In most individuals, type 1 diabetes was diagnosed before the onset of additional AIDs. Despite the youth of the population, 19.2% of individuals with type 1 diabetes developed at least one additional AID, a rate comparable with that found in previous studies [7, 8]. In contrast, only 4.0% of the control group was diagnosed with an AID. The presence of multiple AIDs was notably higher in the type 1 diabetes population. The differences in the distribution of AIDs, particularly the scarcity of individuals diagnosed with atrophic gastritis, may be attributed to the young age of the population and the relatively short follow-up duration compared with previous studies involving older populations [8].

AIDs were more common in female individuals. In Sweden, sex is registered upon birth or on registration as a resident of Sweden, with subsequent acquisition of a personal identity number. The study population was nationwide and population-based and these results on sex differences could be generalised to other populations with a similar composition.

Coeliac disease exhibited the highest prevalence among the AIDs studied. The development of coeliac disease was found to be significantly associated with the age at type 1 diabetes onset, with nearly 20% of children in the youngest age group (0–4 years) being diagnosed. Screening for coeliac disease is of utmost importance within the first 5 years after type 1 diabetes onset since most cases are diagnosed during this period. However, children diagnosed with type 1 diabetes at a young age (0–4 years) were occasionally diagnosed with coeliac disease even after up to 15 years of diabetes duration, suggesting the necessity for considering a screening period longer than 5 years [23]. In Sweden, individuals with type 1 diabetes are screened for coeliac disease at the onset of type 1 diabetes and at least during the first 5 years after onset. The absence of general-population screening implies an underdiagnosis of coeliac disease among people without diabetes [24], thus contributing to the high HR observed for this condition in the diabetes population in this study.

Thyroid disease developed in 7.1% of type 1 diabetes individuals. These findings confirm those of previous studies reporting a prevalence ranging from 3% to 18% in type 1 diabetes populations [4]. Although thyroid disease is more commonly observed in older populations, it was frequently diagnosed even in this young population, emphasising the need for regular screening, ideally annually or every 2 years, for this disease from the onset of type 1 diabetes, as recommended by the International Society for Paediatric and Adolescent Diabetes (ISPAD) guidelines [23]. Thyroid disease could also have been underdiagnosed in the control population in this study, as there is no general-population screening programme after birth.

Children diagnosed with type 1 diabetes during childhood had a significantly higher risk of developing Addison’s disease during adolescence or as young adults compared with the control group. Despite the low number of individuals diagnosed with Addison’s disease, the potential lethality of this condition underscores the importance of physician awareness regarding this risk among individuals with type 1 diabetes.

Vitiligo was found to affect only 0.7% of the type 1 diabetes population. While there is no treatment for vitiligo, its recognition is crucial as it can precede other AIDs, prompting clinicians to be vigilant for signs of conditions such as thyroid disease [13].

Rheumatic joint disease was more prevalent in individuals with type 1 diabetes. Uveitis, a known non-articular manifestation of rheumatic joint disease [25], was also more common in the type 1 diabetes population. Unlike previous studies that suggested the development of juvenile idiopathic arthritis prior to the diagnosis of type 1 diabetes or impaired metabolic control due to steroid treatment [4], our results did not confirm these associations.

Psoriasis developed in 0.9% of the individuals with type 1 diabetes, a lower prevalence than that reported in a small previous study (4.7%) [26], potentially due to the young age of our population.

No increased risk was observed for the development of inflammatory bowel disease, systemic connective tissue disorders or multiple sclerosis in the type 1 diabetes population, possibly due to the relatively short follow-up time and/or the young age of the population. Alternatively, this lack of association could be attributed to a distinct genetic susceptibility for these diseases compared with the other AIDs associated with type 1 diabetes.

The peak appearance of different autoantibodies, such as islet autoantibodies, coeliac and atrophic gastritis autoantibodies and thyroid autoantibodies, in first-degree relatives of individuals with type 1 diabetes [27] complies with the order of clinical onset of the different AIDs in this study.

There is a well-known connection between type 1 diabetes, metabolic control and long-term complications, as well as increased mortality [16, 28]. The burden of disease may further increase with the presence of additional AIDs. Previous smaller studies have reported that blood glucose levels are affected by various AIDs [13]. However, our study did not find any statistically significant impact on metabolic control, consistent with the results of other large-scale studies [7, 8]. Starting from 1 year of diabetes duration and onwards, the mean HbA_1c_ levels of individuals both with and without additional AIDs exceeded the Swedish target for metabolic control (48.0 mmol/mol [6.5%]). By the end of the follow-up period, the mean HbA_1c_ was 62 mmol/mol (7.8%). Although we did not observe increased mortality risk associated with autoimmune comorbidity in this study, it should be noted that the population was relatively young, with the oldest individuals being 37 years old by the end of the study period.

When conducting a register-based study, the data are solely dependent on the quality of the registrations, with limitations resulting from potential inaccuracies due to incorrect registrations. Furthermore, the linking of data to the NPR is contingent upon how clinicians have documented patient diagnoses in the medical records. Additionally, we had no control over which tests and examinations were performed to provide information for the different diagnoses. Another limitation is that we had no access to the family history of AIDs in the included individuals. However, this study’s strengths lie in its large sample size and the use of a control group.

Future studies should aim to provide a more detailed description of each AID associated with an elevated risk in the type 1 diabetes population. Moreover, it would be interesting to conduct a similar study with a longer follow-up time. The risk of long-term complications in individuals with type 1 diabetes and autoimmune comorbidity is not well understood and warrants further exploration. Additionally, studying the association between HLA genotyping and autoimmune diseases, in addition to the well-established connection between HLA type and the development of coeliac disease, could provide valuable insights into the risk of AIDs.

In summary, our study reveals a high prevalence of AIDs, particularly coeliac disease and thyroid disorders, in a young population of individuals with type 1 diabetes. No associations were found between autoimmune comorbidity and impaired metabolic control or increased mortality risk.

## Data Availability

The de-identified individual participant datasets that underlie the results reported in the current study can be obtained from the corresponding author upon reasonable request.

## Abbreviations

AID: Autoimmune disease
CDR: Cause of Death Register
NDR: National Diabetes Register
NPR: National Patient Register
SPDR: Swedish Prescribed Drug Register
